# The regulatory role of long non- coding RNAs as a novel controller of immune response against cancer cells

**DOI:** 10.1007/s11033-022-07947-4

**Published:** 2022-10-07

**Authors:** Dina Mofed, Jihad I Omran, Salwa Sabet, Ahmed A Baiomy, Marwan Emara, Tamer Z. Salem

**Affiliations:** 1 Molecular Biology and Virology lab, Biomedical Sciences Program, UST, Zewail City of Science and Technology, October Gardens, 6th of October City, 12578 Giza, Egypt; 2 Department of Zoology, Faculty of Science, Cairo University, Giza, Egypt; 3 Center for Aging and Associated Diseases, Helmy Institute for Medical Sciences, Zewail City of Science and Technology, 12578 Giza, Egypt

**Keywords:** LncRNAs, Immunotherapy resistance, Immunotherapy response, microRNAs, cancer cells

## Abstract

Immunotherapy has been established as a promising therapy for different cancer types. However, many patients experience primary or secondary resistance to treatment. Immune cells and anti-inflammatory factors are regulated by long noncoding RNAs (lncRNAs). In addition, lncRNAs have a role in immune resistance through antigen presentation loss or attenuation, PD-L1 upregulation, loss of T-cell activities, and activation of G-MDSCs and Tregs in the tumor environment. LncRNAs can also influence the interaction between cancer stem cells and immune cells in the tumor microenvironment, potentially resulting in cancer stem cell resistance to immunotherapy. Immunological-related lncRNAs can influence immune responses either directly by affecting neighboring protein-coding genes or indirectly by sponging miRNAs through various mechanisms. We have emphasized the role and levels of expression of lncRNAs that have been linked to immune cell formation, differentiation, and activation, which may have an influence on immunotherapy efficacy.

## Introduction

**Fig. 1:**
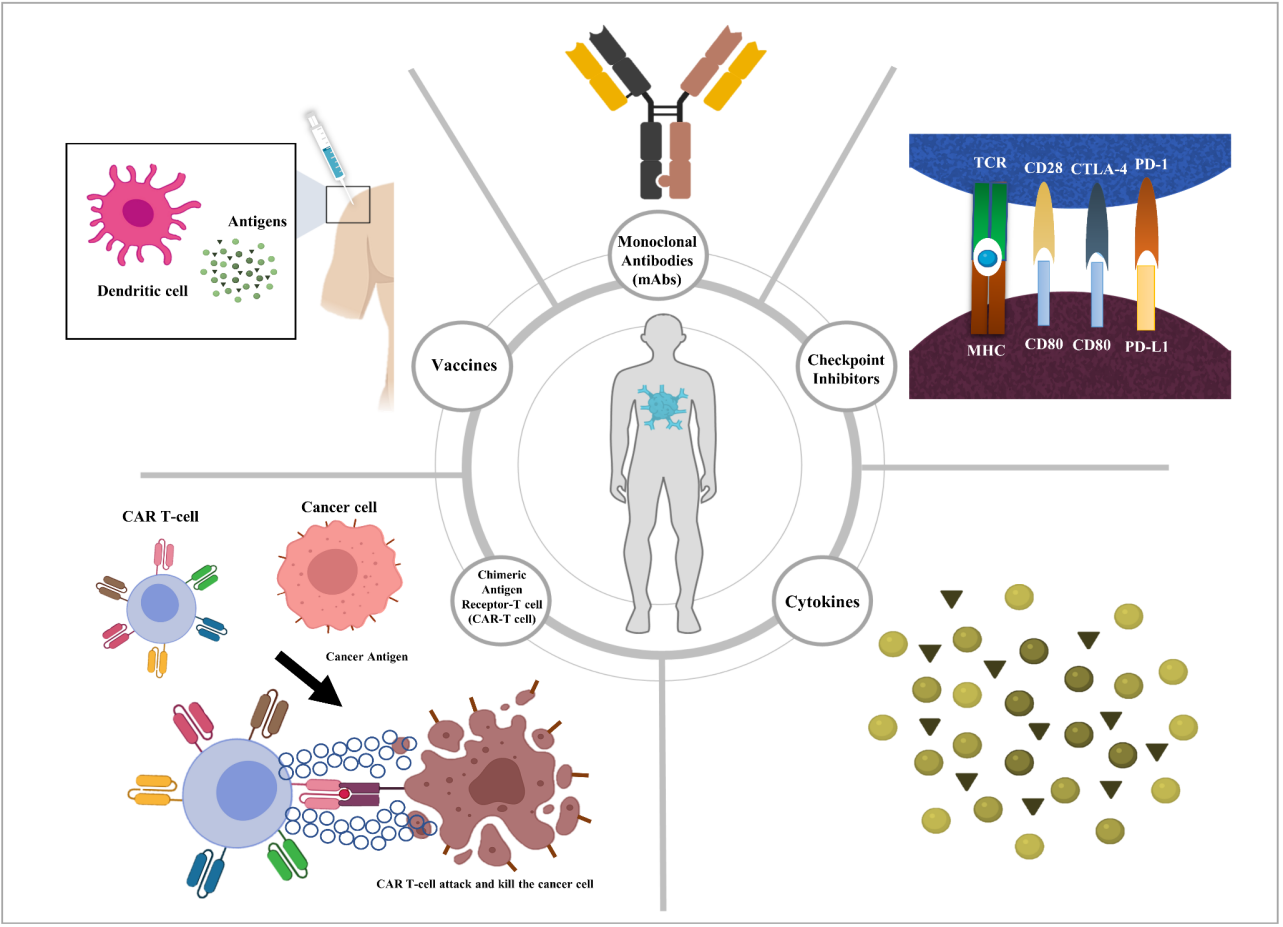
Types of cancer immunotherapies. The figure depicts different cancer immunotherapies, including monoclonal antibodies (mAbs), checkpoint inhibitors, cytokines, vaccines, and chimeric antigen receptor-T cells (CAR-T cells). The figure was created using Biorender (https://biorender.com/)

Cancer and the immune system are bonded strongly together. Immune cells switch from a passive sentinel to an active responder when they detect foreign threats or endogenous alterations in the microenvironment [1]. Cancer immunotherapy (CI) is one of the developing therapy forms which can supplement conventional cancer treatments like chemotherapy, radiation and surgery [2]. Conventional cancer treatments can be destructive to all dividing cells leading to severe side effects like anemia, exhaustion, and emotional stress [3]. However, CI aims to activate the immune system directly to identify and destroy tumor cells and is therefore potentially more specific. The primary characteristics of CI are the broadness of response, specificity and memory against tumor antigens, which can result in better clinical outcomes and consequently improve quality of life, especially in patients with metastatic disease [4]. CI encompasses many different approaches like the use of monoclonal antibodies (mAbs), checkpoint inhibitors (CPIs), cytokines, vaccines, and a chimeric antigen receptor-T cell (CAR-T cell) therapy (Fig. 1) [5]. These approaches can be either active or passive immunotherapies.

In passive immunotherapies, immune checkpoint inhibitors (ICIs) are different monoclonal antibodies which deactivate checkpoint receptors and activate T cells to kill cancerous cells. Anti-PDL1 and anti-CTLA-4 ICIs are passive immunotherapy drugs which have recently been used [6]. Active immunotherapies mainly aim to produce long-lasting memory responses, via the exploitation of immune cells such as dendritic cells which are potent immunomodulatory cytokines and antigen-presenting cells (APC) [7]. However, there are various obstacles to CI which make active and passive immunotherapies highly challenging. The most common challenge is that many individuals fail to respond to CI due to high mutation rates. This suggests that CI must be individualized by carefully comprehending and identifying the rate-limiting step operating on a specific patient [8]. Several cellular mechanisms can affect the functions of the immune system, which could influence the patient’s response to immunotherapy. LncRNAs are one of these strategies. LncRNAs are transcripts that are longer than 200 nucleotides and lack the ability of protein-encoding [9], due to the presence of 7-methyl guanosine (m7G) at their 5′ ends. LncRNAs are not appropriately spliced at the 3’ ends compared to mRNA, which has full splicing at the 3’ end [10]. Although more than 90% of lncRNAs have no protein-coding potential, certain transcripts have been shown to produce short open reading frames (ORFs) of 300 nt or less, which could be translated into small peptides [11].

LncRNAs have a critical role in determining immune responses, therefore they form a logical class of target in cancer immunotherapy studies. Because of their condition-specific expression pattern, lncRNAs are an appealing target for prospective biomarkers and treatments [12]. LncRNAs play a role in immune cells` regulation system; for example; in many particular types of immune cells, the expression of lncRNAs is stimulated by the activation of Toll-like receptor (TLR) when bind with intracellular signalling pathways (such as NF-κB). Also, it can be suppressed by activation of cytokines receptors such as IL-6 or TGF-β, consequently, mediating immune responses [13]. In addition, lncRNAs control cytokines and immune checkpoints and enhance the development of the immunosuppressive environment, which either promotes the progression or suppression of tumor and drug resistance [14]. In this review, we try to summarize the critical roles and functions of these lncRNA-mediated approaches, which have considerable potential in immunotherapies to facilitate cancer treatment and diagnosis.

## The role of lncRNAs in gene expression

**Fig. 2:**
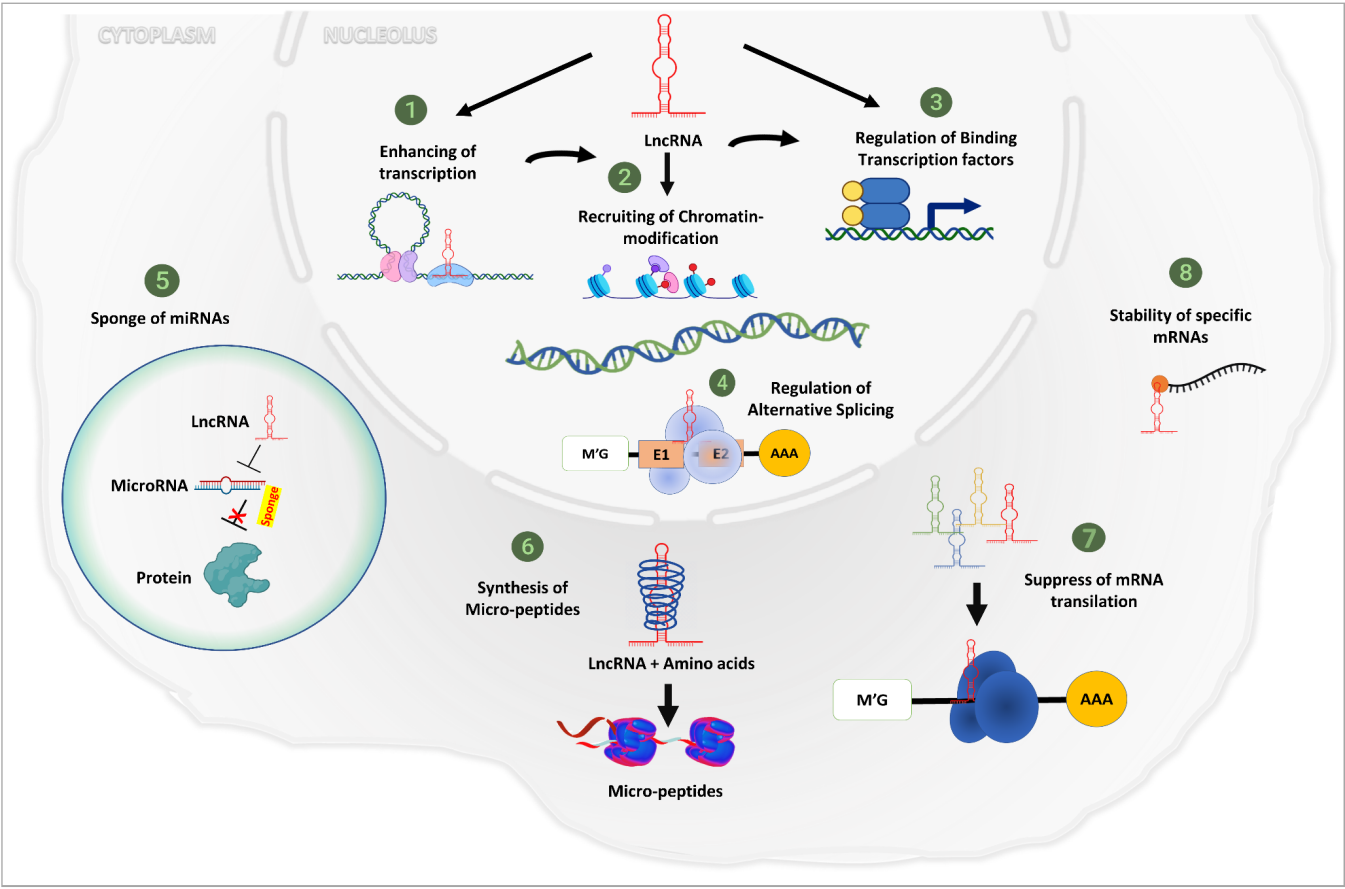
Mechanisms of lncRNAs in the cell. The figure depicts the mechanisms of lncRNAs in the nucleus via (1) enhancing DNA transcription; (2) targeting chromatin-modifying proteins at specified genomic locations; (3) regulating the binding of specific transcription factors; and (4) regulating alternative splicing or in the cytoplasm via (5) acting as a sponge or a decoy for miRNAs; (6) encoding the functional micro-peptides; (7) binding and suppressing the translation of mRNAs; and (8) binding specific mRNAs to enhance stability. The figure was created using Biorender (https://biorender.com/)

LncRNAs have a crucial role in different biological processes, including transcriptional activation and interference, chromatin remodeling, mRNA translation and RNA processing [15] (Fig. 2). Most lncRNAs’ biological activities and molecular mechanisms are still substantially unknown, with only a few being partially understood. Existing evidence suggests that these metabolites serve key roles in the epigenetic, transcriptional, and post-transcriptional control of various cellular activities, including protein-coding gene expression [16].

As previously noted, most lncRNAs preferentially localize in the nucleus and chromatin, and growing evidence suggests that some nuclear lncRNAs epigenetically control gene expression by modifying chromatin structure [17]. LncRNAs mediate changes in chromatin and gene expression in two ways. First, they interact directly with chromatin-modifying enzymes. They act as guides in cis or trans, attracting chromatin modifiers to specific genomic loci to promote DNA methylation or histone modification. These modifications regulate chromatin states and effects on gene expression [18]. In the second mechanism; lncRNAs act as adaptors, connecting specific chromatin loci to ATP-dependent chromatin-remodelling complexes and serving as guides for the regulation of nucleosome remodelling and gene expression [18].

Furthermore, lncRNAs have been discovered to be important regulators of epigenetic processes, including X-chromosome inactivation, genomic imprinting, cellular differentiation, and maintenance of cell identity [19]. LncRNAs regulate gene expression at the transcriptional level by different mechanisms as (A) Gene transcription regulation; they facilitate mobilization of the transcription factors to the promoter region of target genes, interacting with RNA polymerase II, affecting protein localization, and acting as transcriptional activators or repressors. (B) Repression of target gene transcription; they form lncRNA-DNA hybrids with the target gene and promote DNA methylation or acetylation [20]. LncRNAs mediate mRNA stability, translation, degradation, and pre-mRNA alternative splicing genes. Thus they affect gene expression and regulate biological processes at the post-transcriptional level.

LncRNAs function as competing for endogenous RNAs (ceRNAs) or sponges, decoys, signals, guides and scaffolds according to their mode of action [21]. LncRNAs act as ceRNAs or endogenous microRNAs (miRNAs) sponges through two pathways; (A) they can sequester miRNAs, preventing them from attaching to target mRNAs. (B) In the case in which lncRNAs and miRNAs have common binding sites in the target mRNA, lncRNAs can interact directly with target mRNA transcripts, blocking miRNA binding sites in mRNA molecules. Interactions between miRNAs and ceRNAs are important for regulating many basic biological functions [22].

Decoy lncRNAs act as modulators of transcription by trapping or decreasing the availability of different regulatory factors, including catalytic proteins, miRNAs, transcription factors and sub-units of larger chromatin-modifying complexes [23]. Scaffold lncRNAs have a vital structural role, they serve as platforms for the assembly of multiple-component complexes, and other regulatory co-factors such as ribonucleoprotein (RNP) complexes. RNP complexes can regulate gene expression by targeting certain genomic regions [24]. Guide lncRNAs can regulate transcription by binding with different factors like regulatory or enzymatically active proteins and organize their localization on specific sites on the genome either cis (adjacent) or trans (distant) from their transcriptional locus [25]. Signal lncRNAs function as molecular signals to control transcription in response to different stimuli. As a result, the presence and production of these components act as an indicator for transcription activity and also under specific conditions, they can modulate specific signaling pathways [26].

In summary, lncRNAs are widely expressed and play important roles in gene regulation. They can modulate chromatin function, regulate the assembly and function of membraneless nuclear bodies, modify the stability and translation of cytoplasmic mRNAs, and interfere with signaling pathways depending on their localization and specific interactions with DNA, RNA, and proteins.

## The effect of lncRNAs on the efficacy of cancer immunotherapy

**Fig. 3:**
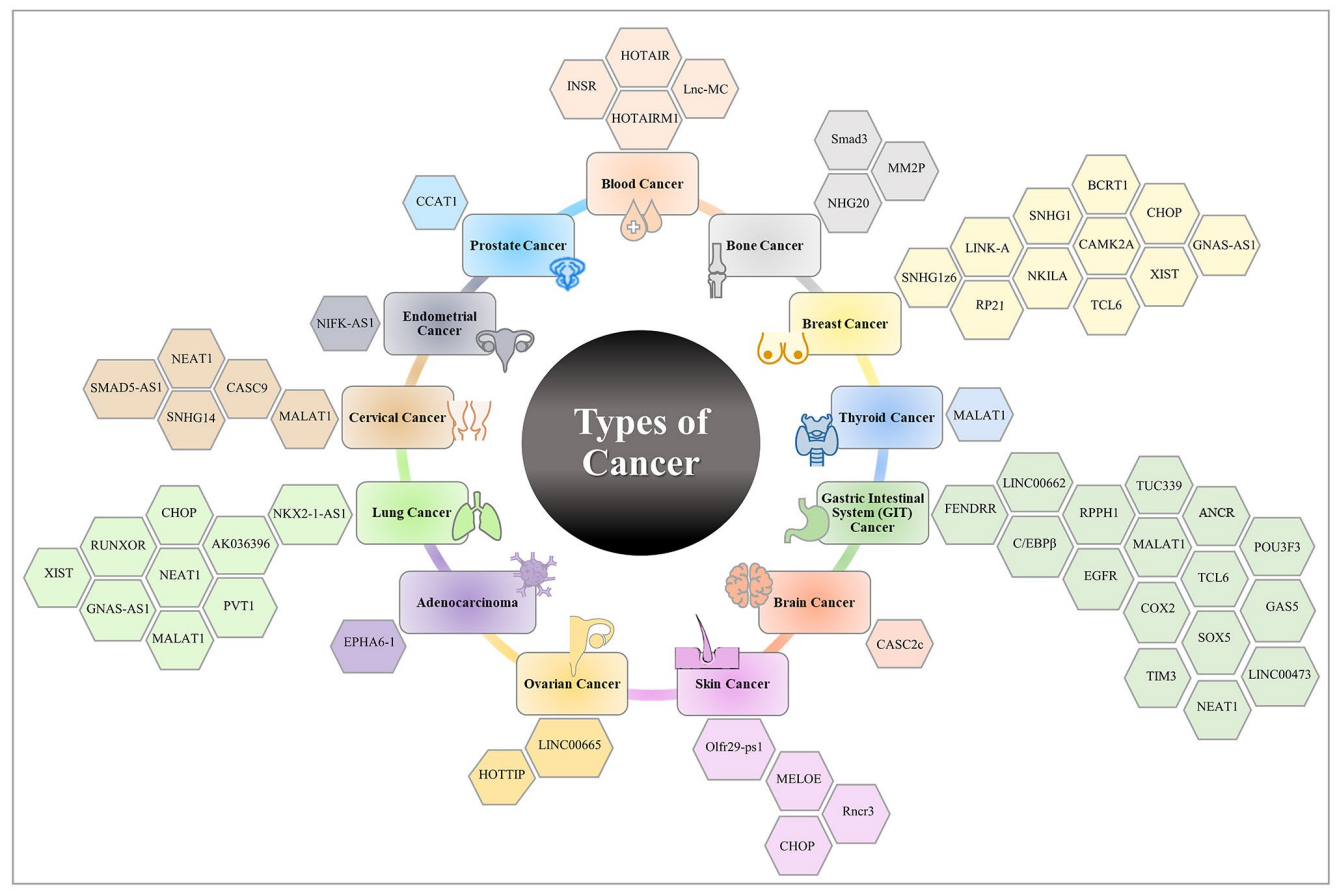
LncRNAs are involved in different types of cancer. The figure depicts the lncRNAs involved in different types of cancer. Each type of cancer is designated with a different color code, while LncRNAs are presented in hexagon shapes

Rapid advances in transcriptomic profiling have led to the identification of immune-related lncRNAs as regulators of immune cell-specific gene expression that mediates both immune stimulation and suppression, implying that lncRNAs could improve the efficacy of immunotherapy against tumors. Tables 1 and 2 summarize the regulatory role of lncRNAs in cancer progression or cancer suppression via different immune responses. Some lncRNAs act as immunomodulators, influencing inflammatory cytokine production, altering innate immune cells, or creating resistance to immunotherapy [27]. Others can affect antigen presentation, control the production of PD-L1, and regulate the actions of immunosuppressive cells to modify the T-cell-mediated immune response that leads to immunosuppression [28]. In this review, we focus on lncRNAs associated with the immune response as a target for improving immunotherapy resistance in different cancer types (Fig. 3). For Example, in acute lymphoblastic leukemia (ALL), lnc- Insulin receptor (INSR) has a role in tumor growth and progression via overexpression of T regulatory cells (Treg) and decreases the percentage of cytotoxic T lymphocytes (CTLs) [29]. In addition, lnc INSR directly attaches and blocks INSR ubiquitination, leading to activation of INSR and PI3K/AKT-signaling pathway, which results in induction of suppressive immune microenvironment (IME).

**Table 1:** The role of lncRNAs in enhancing cancer progression by different immune pathways

No	Type of cancer	Name of lncRNA	HGNC ID number	Model of study	Samples origin	Pathway	Immune effect	Ref
1.	Acute promyelocytic leukaemia	HOTAIRM1	37,117	*In vitro* and clinical study	cell lines: NB4, HL-60, K-562, THP1, Jurkat, SuDHL6, HEK293, HeLa, HepG2, Saos2, and human leukocyte cells	Promote the transcription of the myeloid differentiation genes CD11b and CD18	Induce differentiation and promote the functions of the myeloid cells, resulting in enhance immunogenicity	[69]
2.	Leukemia	HOTAIR	33,510	In vivo study	Inbred mice with leukaemia DBA/2 cell line	Inhibition of NK cell activity and the ratio of CD4/CD8 in B-lymphocyte, increase in activation of Wnt/β‐catenin signalling pathway	Immunologic rejection in leukaemia	[31]
3.	Acute Lymphoblastic leukaemia (ALL)	INSR	6091	In vivo and clinical study	Clinical samples and animal model NOD/SCID mice	Overexpression of Tregs levels and decrease in cytotoxic T-lymphocyte percentages	Enhance immunosuppressive environment which has a role in cancer progression	[29]
4.	Breast and lung cancer	NKILA	51,599	Clinical study	Human Tumor samples and peripheral blood samples	Increase sensitivity of tumor-specific cytotoxic T-lymphocytes (CTLs) and type 1 helper T-cells (TH1) to Activation-induced cell death AICD by inhibiting NF-κB activity	Increase tumor immunological escape	[70]
5.	Breast cancer	CAMK2A	1460	In vivo and clinical study	Tissue samples and animal models; Female BALB/c nude mice and cell lines; MDA-MB-23 and MDA-MB-468 and HEK293Tand HUVEC	Activate NF-κB signalling by regulating Ca^2+^/calmodulin-dependent kinase PNCK, which in turn phosphorylates IκBα	macrophage infiltration, angiogenesis, tumor microenvironment remodeling, and cancer progression	[71]
6.		P21	13,434	*In vitro* study and in vivo study	Animal models; BALB/c mice and PyVT-MMTV mice and cell line; Mouse breast cancer cell line 4T1 and Lewis lung carcinoma (LLC)	Overexpression of LncRNA –p21 in BC inhibits p53 by enhancing MDM2 to antagonize p53 and suppress NF-κB and STAT3 pathway	Suppress macrophage polarization into pro-inflammatory macrophages	[33]
7.	Thyroid cancer	MALAT1	29,665	*In vitro* study and clinical study	Human peripheral blood mononuclear cells (PBMCs) and thyroid cell lines: SW1736, KAT18, FTC133, and HGC-27	MALAT1 modulates FGF2 protein secretion from TAMs and suppresses inflammatory cytokines release	Accelerate proliferation, migration, and invasion of tumor cells and angiogenesis	[72]
8.		SOX5	11,201	*In vitro* study and clinical study	Human samples, colorectal cancer cell line: Caco-2 and Mouse colorectal cancer cell line: MC-38	Downregulation of indoleamine 2,3-dioxygenase 1 (IDO1) results in regulation of the infiltration and cytotoxicity of CD3^+^CD8^+^T cells	Unbalanced in the tumor environment, which leads to cancer progression and proliferation	[73]
9.	Colorectal cancer (CRC)	RPPH1	19,273	*In vitro* study and clinical study	Patient samples and cell lines; HCT8, SW620, HT29, and human embryonic kidney 293T	Upregulates TUBB3 which acts as a drug resistance marker by inhibiting its ubiquitination. In addition, increase exosomes-mediated macrophages M2 polarization	Promote metastasis and proliferation of CRC	[74]
10.		EGFR	3236	*In vitro* study and clinical study	Blood or tissue samples and cell lines: 97 H, and Huh7	Enhance Tregs (CD4^+^CD25^+^Foxp3^+^) which have a role in down-regulation of the proliferation of effector T-cells	Enhance immunosuppressive state and immune escape in HCC	[35]
11.	Hepatocellularcarcinoma (HCC)	Lnc-Tim3	18,437	Clinical study	Patient samples	Upregulation of Inc-Tim3 is linked to downregulation of IFN-γ and IL-2 in tumor-infiltrating CD8 T-cells of HCC patients	Lnc-Tim3 increases T-cell exhaustion, a phenotype that is associated with compromised anti-tumor immunity	[75]
12.		ANCR	482	*In vitro* study and clinical study	Patient samples and cell lines: THP-1 cells and human gastric cancer cells: HGC-27	Overexpression of lncRNA ANCR in GC suppress polarization of macrophages to M1 via FoxO1 downregulation	Decrease M1 macrophage polarization and enhance invasion and metastasis of GC cell	[76]
13.	Gastric cancer (GC)	POU3F3	9216	Clinical study	Patients with gastric cancer, healthy donors and PBMCs were separated from patients	Elevate the differentiation of Tregs (CD4^+^CD25^+^Foxp3^+^) which have a role in activating of TGF-beta signal pathway	Increase cell proliferation, and cancer progression and may have a role in immunosuppression	[77]
14.	Lung cancer	Lnc- chop	2726	*In vitro* study and in vivo study	Different Mice models and cell lines: B16, 4T1, and HEK293T	Association between lnc-chop with both inhibitory proteins (CHOP and the C/EBPβ isoform leads to activation of C/EBPβ and upregulation of the expression of arginase-1, NO synthase 2, NADPH oxidase 2, and cyclooxygenase-2	Decrease immune functions of Myeloid-derived suppressor cells (MDSCs)	[78]
15.		PVT1	9709	Ex vivo study	C57BL/6 mice were injected with LLC cells	HIF-1α modulates the expression of Pvt1 under hypoxia, Pvt1 activates Myeloid-derived suppressor cells (MDSCs) which have a role in blocking T-cell-induced antitumor responses	Enhance immunosuppression activity in lung carcinoma	[39]
16.		RUNXOR	-----	Clinical study	Lung cancer patients	Downregulation of tumor suppressor protein RUNX1 by direct binding to its promoter, which has a role in the regulation of Myeloid-derived suppressor cells (MDSCs	Acceleration of immunosuppression activity, lung cancer progression	[79]
17.		NEAT1	30,815	*In vitro*, In vivo and clinical study	Lung cancer tissues, different mice models and human lung cancer cell lines; A549, HCC1299, HCC827, NCI-H460, SK-MES-1 and NCI-H358	Inhibition of cytotoxic T-cell by direct interaction of lncRNA-NEAT1 and DNMT1 through cGAS/STING pathway	Evasion of T-cell tumor immunity and cancer progression	[80]
18.		XIST	12,810	*In vitro* study	Human cell lines; 293T-cells, THP-1 and A549	TCF-4, which acts as a nuclear response, directly interacts with lncRNA XIST and induces the expression of IL-4	Regulate M2 polarization through IL-4	[81]
19.	Ovarian cancer (OC)	HOTTIP	37,461	*In vitro* study and clinical study	Patient samples and cell lines: 293 T-cells and SKOV3, OVCAR3, and Hy-A8	Upregulation of HOTTIP is associated with an increase in IL-6 via binding with c-jun. that leads to overexpression of PD-L1 expression	Inhibit T-cell proliferation and acceleration of tumor immune evasion	[82]

**Table 2:** The role of lncRNAs in suppression of cancer progression by different immune pathways

No.	Type of cancer	Name of lncRNA	HGNC ID number	Model of study	Sample origin	Pathway	Immune effect	Ref
1.	Triple-negative breast cancer (TNBC)	LINK-A	27,924	Ex vivo Study	Transgenic mouse model cell lines	Downregulation of PLC compounds (TPSN, TAP1, TAP2, and CALR), suppress CD8 + T cell infiltration and MHC I complex	Loss of antigenicity and intrinsic tumor suppression provides a new strategy for immunotherapy	[83]
2.	Breast cancer	TCL6	13,463	Clinical study	Human samples	Up-regulation of lncRNA, TCL6 is strongly associated with overexpression of PD-1, PD-L1, PD-L2, and CTLA-4 through JAK/STAT cascade and Regulation tumor-associated B-cells, CD8 + T-cells, CD4 + T-cells, neutrophils, and DCs	Immune infiltration, suppression of the immune checkpoint blockade (ICB) immunotherapy-related genes (PD-1, PD-L1, PD-L2, and CTLA-4)	[32]
3.	Colorectal cancer (CRC)	C/EBPβ	1834	*In vitro*, in vivo and clinical studies	Patient samples, different mice models, and different cell lines: B16, LLC, 4T1, MCF-7, MDA-MB, lymphoma U937, HT-29, and HEK 293T	Direct binding of lnc-C/EBPβ with transcription factor C/EBPβ inhibits the immunosuppressive function of MDSCs where transcription factor C/EBPβ plays an important role in regulating MDSCs functions	Limiting over suppression of MDSCs on immune response	[84]
4.	Hepatocellular carcinoma (HCC)	COX2 (PTGS2)	9605	*In vitro*, in vivo and clinical studies	Different mice models, peripheral blood and tissue samples and cell lines; B16, LLC, 4T1, Hek293T, MCF-7, MDA-MB, lymphoma U937, Hela and HT-29	Enhance the polarization of M1 macrophages and inhibiting the polarization of M2 macrophages	Suppress the immune evasion, invasion, and migration of HCC cells	[34]
5.	Lung cancer	NKX2-1-AS1	40,585	*In vitro* study	Human lung carcinoma cell line: NCI-H441, NCI-H661, NCI-H1299, Calu-6, and BEAS-2B	Negatively regulates endogenous CD274/PD-L1	Limiting motility and immune system escape of lung carcinoma cells	[38]
6.	Lung cancer	MALAT1	29,665	Clinical study	Peripheral blood mononuclear isolated from patients and healthy donors	Downregulation of MALAT1 is associated with the increase of MDSCs. MDSCs increase the secretion of ARG-1 which inhibits T-cell activation. In addition, MDSCS induces COX-2 which relates to the decrease in the quantity and functions of CD8^+^T-cells	Inhibit tumor immune escape, tumor development, and tumor progression	[85]
7.	Melanoma	MELOE	----	*In vitro* study	Different cell lines; Meso34 and Meso61 and SW707	Induce the translation of immunogenic antigens (MELOE-1, MELOE-2, and MELOE-3) via an IRES-dependent mechanism	MELOE-3 induces immunogenicity in melanoma cells compared to others (MELOE-1, MELOE-2), so it has a potential effect as T cell target for melanoma immunotherapy.	[86]

PI3K is an abundant protein in white blood cells and abnormality in its signaling pathway inhibits T cell activation. Also, the PI3K/AKT pathway enables T cell receptor signaling to control the expression of forkhead box P3 (Foxp3), which binds to certain areas of DNA and helps to regulate the activity of genes involved in immune system regulation [30]. LncRNA HOTAIR increases the immunologic rejection in mice leukaemia cells via the activation of the Wnt/-catenin pathway, in which mice given HOTAIR mimics as well as small interfering RNA HOTAIR. The results revealed the up regulation of lncRNA HOTAIR, which resulted in an increase in the percentage of leukocytes and decreasing the percentage of PLTs and haemoglobin concentration. Furthermore, increased HOTAIR suppressed Ig synthesis, natural killer (NK) cell activity, and the ratio of CD4/CD8 T cell subsets in B-lymphocytes, and lowered the levels of transforming growth factor, interferon, interleukin-10, and tumor necrosis factor in PB and T-lymphocyte proliferation. Additionally, over-expressed HOTAIR boosted the expression of cyclinD1, GSK-3, and c-Myc in the bone marrow of mice, resulting in better survival and proliferation [31].

In breast cancer, lnc- T-cell leukemia/lymphoma 6 (TCL6) correlated with immune infiltration and is therefore considered a useful prognostic molecular marker. Low TCL6 expression is linked to a poor prognosis, particularly in progesterone receptor-negative (PR) and luminal B breast cancer patients. Additionally, the lncRNA TCL6 may influence immune-related pathways, including JAK/STAT. TCL6 regulates tumor-associated B cells, CD8 + T cells, CD4 + T cells, neutrophils, and Dendritic cells (DCs). Furthermore, TCL6 is associated with tumor-infiltrating lymphocytes (TILs) and immunological checkpoint markers, including PD-1, PD-L1, PD-L2, and CTLA-4 [32].

LncRNA p21 plays an important role as a regulator of tumor-associated macrophages (TAMs) in breast cancer. TAMs show different phenotypes in cancer development, where they have a pro-inflammatory phenotype, generate an anti-tumor type-1 inflammatory response, and suppress tumor cell proliferation by generating TNF, reactive oxygen species (ROS), or phagocytosis during the primary stage of tumor development. In contrast, tumor microenvironment-educated macrophages release IL-10 and TGF and inhibit the activation of CTLs and NK cells at advanced stages of tumor development. Downregulation of lncRNA p21 facilitates interaction between p53 and Mouse double minute 2 homolog (MDM2), resulting in activation of NF-κB and STAT3 signaling pathways, reversed TAMs phenotype and generating TNF-α to destroy cancer cells [33].

LncRNA Cyclooxygenase-2 (Cox-2) is located about 50 kb downstream of the mouse Cox-2 gene. LncRNA Cox-2 is a broad-acting regulatory component of the inflammatory response regulation circuit, where the repression or activation of various immune genes can mediate by it. In hepatocellular carcinoma (HCC), lncRNA Cox-2 has an important role in the suppression of the tumor immune evasion and development by enhancing the polarization of M1 macrophages and suppressing the polarization of M2 macrophages [34]. In contrast, the lnc-epidermal growth factor receptor (EGFR) enhances HCC progression and development. LncRNA EGFR enhances differentiation of Treg and suppresses CTLs by binding with EGFR and blocking its interaction with c-Casitas B-lineage Lymphoma (c-CBL). The latter is an E3 ubiquitin-protein ligase that helps in cell signaling and protein ubiquitination. Additionally, lncRNA EGFR has a positive feedback loop in Treg, where it increases the expression of EGFR resulting in activation of the expression of its downstream extracellular signal-regulated kinases 1/2 (ERK1/2), Activator protein 1 (AP-1) and Foxp3 expression, which induce immunosuppression in HCC patients [35].

In a recent *in silico* study, Xia P. and colleagues used a similar strategy with The Cancer Genome Atlas (TCGA) and Chinese Glioma Genome Atlas (CGGA) patients. A total number of 812 immune-related lncRNAs founded to be linked directly to glioma. The authors developed a risk score formula using Cox regression and the least absolute shrinkage and selection operator (LASSO) analysis to investigate the differences in overall survivals (OSs) between high- and low-risk groups. The risk score (RS) method contained eleven immune-related lncRNAs that were linked with survival. There is a marked relation between high-risk score cases that had poor survival in both the TCGA and CGGA groups related to Glioma patients. The RS formula exhibited a good prediction accuracy in the CGGA dataset equals (5-year Area under Curve (AUC) = 0.730) and could accurately forecast the prognosis of glioma patients that equals (5-year AUC = 0.749). These results from the model’s robust prediction function, give specific guiding values for glioblastoma etiology and clinical treatment analysis, allowing users to find prospective therapeutic targets for glioma treatment [36].

In another study, the immune-related lncRNA signature (IRLS) in bladder cancer was determined using the LASSO Cox regression model. Using LASSO Cox regression analysis, the authors chose five prognostic lncRNAs to create IRLS. They discovered that IRLS positively linked with immune cell infiltration and expression of important immunological checkpoints in the tumor microenvironment (TME), implying that immunosuppressive TME may play a role in poor prognosis [37]. LncRNA NKX2-1-AS1 was originally detected in lung carcinoma cells. Overexpression of lncRNA NKX2-1-AS1 can reduce cell migration and immune system evasion by decreasing the expression of CD274, the gene encoding PD-L1 [38]. In contrast, lncRNA plasmacytoma variant translocation 1 (Pvt1) induces lung cancer progression and development, where HIF-1α regulates the expression of Pvt1 under hypoxia. Pvt1 activates Myeloid-derived suppressor cells (MDSCs) which have a role in blocking T-cell-induced antitumor responses resulting in induction of immunosuppression activity in lung carcinoma [39].

In summary, lncRNAs are expressed in a variety of immune cells and have a role in both innate and adaptive immunity. The discovery of lncRNAs has provided a unique perspective for investigating the regulation of TME. LncRNAs promote the formation of an immunosuppressive microenvironment through related different pathways, like the PI3K/AKT pathway, Wnt/-catenin pathway, JAK/STAT pathway and TNF-α/ NF-κB pathway, thereby controlling the escape of tumors from immune surveillance and promoting the development of metastasis and drug resistance in different cancer types.

## The role of lncRNAs in cancer stem cell resistance to immunotherapy

The cancer immune-editing theory and the hierarchy model of cancer initially explained the ability of cancer stem cells (CSCs) to resist immunological destruction. Pluripotent and long-living cancer cells are subpopulations of CSCs which are responsible for drug resistance, recurrence, and metastasis and tumor initiation [40]. CSCs can hide from the immune-mediated destruction and interact with different immune cells by releasing several intracellular, soluble and membrane-bound factors that allow them to survive and inhibit innate and adaptive anti-tumor immune responses in their niche [41]. The CSCs can dysregulate the expression of various anti-apoptotic proteins, including survivin, Bcl-xL, and Bcl-2 in different tumor types. Also, CSCs can evade immune-mediated apoptosis promoted by NK and effector T cells, or immunotherapeutic antibodies by secreting many cytokines and other molecules, such as IL-10, IL-6, IL-4, FasL, and prostaglandin E_2_ (PGE2). In addition, CSCs maintain the immunosuppressive TME and enhance tumor infiltration by releasing diverse immunosuppressive cytokines and chemotactic factors such as TGF-β, IL-10 and IL-2. These factors otherwise suppress the activities of effector immune cells such as NK cells, CTLs and T cells or/and enhance immunosuppressive immune cell subpopulations, including MDSCs, Tregs and TAMs [42]. Also, other studies on multifaceted interactions between CSCs and the immune system showed that CSCs can recruit and enhance the properties of macrophages into TAMs. TAMs usually express M2 phenotypes which have pro-tumor and immunosuppressive properties and are related to cancer progression and recurrence [43]. TAMs are required for CSCs self-renewal and maintenance in different cancer types via activation of the STAT3/NF-*κ*B pathway (Sainz et al., 2016). DCs are APCs that generate innate or adaptive immune responses. CSCs can recruit immunosuppressive properties of DCs by producing immunosuppressive cytokines, including IL-13, IL-10, IL-4, and co-inhibitory molecules like B7-H3, IDO1 and PD-L1 which induce tumor properties of DCs [44].

The MDSCs play a crucial role in enhancing an immunosuppressive environment in TME and are employed as a prognostic biomarker for patients` response to immunotherapy and their rate of survival. The MSDCs can be categorized into two subtypes based on their different nuclear morphologies: granulocytic-MDSC (gMDSC) and monocytic-MDSC (mMDSC). They have two different immunosuppressive mechanisms. Cross linking between CSCs and MDSCs at the tumor site can enhance tumorigenesis and metastasis in different cancer types. For example; CSCs activate IL-6/STAT3 signaling, which enhances the differentiation of monocytes to MDSCs in breast cancer (Welte et al., 2016). Meanwhile, in ovarian cancer, MDSCs promote the stem-like features of CSCs by increasing the expression of (PGE2) and PD-L1 [45].

In HCC, MDSCs move to the tumor site via ENTPD2/CD39 L1 signaling under hypoxic conditions where liver CSCs are abundant. These MDSCs promote HCC development and decrease the efficiency of PD1 treatment (Chiu et al., 2017). Tregs are CD4 + T cells that promote tumor growth and are usually identified by the Foxp3 + CD25 + CD4 + T cell subset. They suppress effector T cells and other immune cells by secreting immunosuppressive cytokines such as IL-35, IL-10, and TGF- β. The interplay between CSCs and Treg in TME, suggests their roles in maintaining immunosuppression and promoting tumor infiltration of different types of cancers. Mainly, Treg infiltration is induced by CSCs in the TME via co-stimulatory molecules and STAT3 signaling, whereas Tregs regulate CSCs proliferation and expansion either directly through IL-17 and PGE2 production or indirectly through TGF-mediated angiogenesis and epithelial-mesenchymal transition (EMT) [44].

NK cells represent a subpopulation of cytotoxic lymphocytes, known as large granular lymphocytes (LGL) which account for 5–20% of all circulating lymphocytes in humans. NKs are critical for innate immune response and are considered one of the cell types which has the most potential for targeting and killing the tumor cells. CSCs can evade NK by increasing the expression of MHC-I on their surface, where most immune responses are triggered by NK in tumor cells through down-regulation of MHC-I. Also, EMT-derived NK cell is one of the cellular pathways which NK cells promote in CSCs according to the type of cancer. For example; in melanoma, NK cells secret IFN-γ and TNF-α and enhance CSCs to undergo EMT, inducing them to invasive phenotypes and increasing the expression of stemness markers [46].

In contrast, in colorectal cancer, EMT promotes anti-tumor immune response by increasing the expression of NK group 2, member D (NKG2D) receptor which has an important role in cancer immunosurveillance [47]. LncRNAs have a potential impact on immune modulation and regulation of CSCs. Several studies showed that TME and CSCs niches have a crucial role in the maintenance of lncRNA-regulated therapy resistance in CSCs. For example, in HCC and breast cancer, Hypoxia-associated lncRNA (HAL) and lncRNAs runt-related transcription factor 1 Intronic Transcript 1 (RUNX1-IT1) enhance cancer stemness and increase resistance to apoptosis, under the regulation of Hypoxia which represents one of the key features of TME [48, 49]. As mentioned above, crosslinking between CSCs and two pathways- TNF-α/ NF-*κ*B signaling and IL-6/STAT3- regulates the differentiation of many immune cells in TME, promoting immunosurveillance and cancer progression.

LncRNA down-regulated in liver cancer stem cells (lnc-DILC) has an inhibitory role in liver cancer stem cells’ self-renewal and suppresses its expansion by down-regulation of IL-6 transcription and STAT3 activation. In addition, lnc-DILC modulates the crosstalk between TNF-α/ NF-*κ*B signalling and IL-6/STAT3, resulting in a decreased hepatic inflammation and liver cancer stem cell proliferation [50]. Another study showed that crosslinking between lncRNA H19 and TAMs in EMT induced stemness and accelerated HCC invasion of HCC cells *in vitro* through modulation and activation of the miR-193b/MAPK1 axis, suggesting that lncRNA H19 could be a promising anti-cancer therapy strategy for reducing HCC aggression and improving clinical outcomes [51].

In glioblastoma, stem-like subpopulation cells which are exited in multiform and have a role in tumor progression, resistance to apoptosis and recurrence are called glioma stem cells (GSCs). Crosstalk of the miR-146b-5p/HuR/lincRNA-p21 axis inhibits expression and activity of β-catenin, resulting in increased apoptosis and radio sensitivity, decreased cell viability, neurosphere formation capacity and stem cell marker expression, and induced differentiation in GSCs [52]. LncRNA HOTAIR regulates proliferation, invasion, colony formation, and self-renewal capacity through the modulation of the miR-34a/ Sox2/ p53 /p21 axis in breast cancer CSCs [53].

In summary, CSCs release a variety of immunosuppressive cytokines and chemotactic factors such as; PGE2, IL-10, TGF-β, IL-4, IL-13 and IL-35 that affect different immune cells in the TME and promote tumor invasion and progression. Also, the interaction between lncRNAs, CSCs and immune cells in TME, has a key role in immune escape and resistance to immunotherapies through different cellular pathways including; EMT, IL-6/STAT3 signaling pathway, STAT3/NF-*κ*B pathway and TNF-α/ NF-*κ*B pathway.

## The effect of lncRNAs on the miRNAs involved in immunotherapy responses

The miRNAs are a subset of noncoding RNA with a length of ~ 22 nucleotides that have a functional role in the posttranscriptional regulation of protein-coding genes by mRNA cleavage, direct translational suppression, and/or mRNA instability. LncRNAs affect miRNAs through a variety of pathways, as mentioned above, which control a variety of biological processes. The interaction between miRNAs and lncRNAs can influence tumor growth, invasion, and metastasis by enhancing the activation of oncogenic pathways and reducing the expression of tumor suppressors or vice versa [54].

The relationship between lncRNAs and miRNAs was shown to alter cancer immunotherapy resistance in several earlier investigations (Tables 3 and 4). For Example, in breast cancer, overexpression of lncRNA small nucleolar RNA host gene 1 (SNHG1) could decrease immune escape and tumor progression by enhancing the expression of miRNA- 448 and repression of Indoleamine 2,3-dioxygenase (IDO) level, resulting in inhibition of Treg differentiation. The IDO is a vital component of the immune system that aids in natural defense against a variety of infections. Also, it has a role in the suppression of T cell immunity and promoting the maturation and differentiation of Treg cells [55]. In contrast, another study by Liang and his colleagues showed that knockdown of lncRNA breast cancer-related transcript 1 (BCRT1) could decrease breast cancer progression *in vitro* and in vivo. LncRNA BCRT1 acts as a ceRNA for miRNA-1303 which targets Poly pyrimidine tract-binding protein 3 (PTBP3), thus protecting PTBP3 from degradation and promoting breast cancer progression. PTBP3 is an essential RNA-binding protein that regulates gene expression and influences the biological behaviour of many malignancies. It also plays a key role in RNA alternative splicing. In addition, lncRNA BCRT1 can transfer to macrophages by exosomes, accelerating M2 polarization and promoting its impact on tumor progression [56].

**Table 3:** The regulatory role of lncRNAs on miRNAs involved in cancer progression by different immune pathways

No.	Cancer type/origin	Name of lncRNA	HGNC ID number	Model of study	Sample origin	Affected miRNA	Pathway	Immune effect	Ref
1.		MALAT1	29,665	*In vitro* study and clinical study	Patient samples and cell lines; Human DLBCL cell line OCI-Ly10	miR-195	MALAT1 upregulates PD-L1 through the sponge and inhibit miR-195 which has a role in regulating the expression of PD-L1	Increase proliferation,, migration, and immune escape in DLBCL	[87]
2.	Diffuse large B-cell lymphoma (DLBCL)	NEAT1	30,815	*In vitro* and clinical study	Patient samples and cell lines; OCI-Ly1, OCI-Ly8, OCI-Ly10, and SUDHL-4	miR-34b-5p.	MYC modulates the transcription of NEAT1 which acts as a competing endogenous of miR-34b-5p.	Modulate DLBCL proliferation and attenuate apoptosis through the miR-34b-5p-GLI1 pathway	[88]
3.		SNHG14	37,462	*In vitro* study and clinical study	Patient samples and cell lines; GM12878 and293T and A20 andOCI-LY7, DB, U2932, and FARAGE	miR-5590-3p	SNHG14 up-regulates transcription factor ZEB1 by sponge miR-5590-3p and that promotes expression of PD-L1	Enhance diffuse large B cell lymphoma progression and immune evasion through regulating PD-1/PD-L1 checkpoint	[59]
4.	Breast cancer	SNHG16	44,352	*In vitro* study and clinical study	Patient samples and cell lines; MCF-10 A, MCF-7, T-47D, MDA-MB-231 and HEK293	miR-16–5p	BC-derived exosomes transmit SNHG16 which directly sponge miR-16–5p and that induce SMAD5. SMAD5 has a role in the overexpression of CD73 in Vδ1 Treg cells	Activate TGF-β1/SMAD5 pathway and CD73 + γδT1 immunosuppressive effect	[89]
5.	ER^+^ breast cancer	GNAS-AS1	24,872	*In vitro* study and clinical study	Patient samples and cell lines ; MCF-7, T47D, MCF10A, and THP-1	miR-433-3p	GNAS-AS1 directly inhibits miR-433-3p and suppress the expression of transcription factor GATA3 which regulates M2-type macrophage polarization	Induce M2 macrophage polarization, immunosuppression, and tumorigenesi	[90]
6.	Pancreatic cancer (PC)	SBF2-AS1	27,438	*In vitro* study	cell lines; (PANC-1, BxPC‐3, SW1990, Capan‐2) THP‐1	miR-122‐5p	Up-regulation lncRNA, SBF2-AS1 in M2 macrophage exosomes inhibits the miR‐122‐5p expression, and that leads to increase expression of XIAP	Increase polarization of M2 macrophage-derived exosomes and increase PC progression	[91]
7.		LINC00473	21,160	*In vitro* study and clinical study	Patient samples and cell lines; SW-1990, Panc‐1, BxPC‐3, AsPC‐1, CAPAN‐2 and H6C7	miR-195‐5p	Overexpression of PD-L1 through the sponge and inhibit miR-195‐5p	Inhibit CD8^+^ T cells activation and enhance immune evasion	[92]
8.	Hepatocellular Carcinoma (HCC)	LINC00662	27,122	*In vitro* study	Cell lines; QSG-7701, HCCLM3, MHCC97H, Huh7, and SK‐HEP‐1, and THP‐1	miR-15a, miR‐16, and miR‐10	Promote tumor-derived WNT3A and activating Wnt/β-catenin signalling in macrophages by competitively binding miR-15a, miR‐16, and miR‐10	Enhance M2 macrophage polarization, HCC cell proliferation, cell cycle, and tumor cell invasion and repress HCC cell apoptosis.	[58]
9.		MALAT1	29,665	*In vitro* study and clinical study	Patient samples and cell lines; LO2, HepG2 Huh-7 THP-1 and (HUVECs)	miR-140	UP regulation of MALAT1 in HCC inhibits VEGF-A expression via direct sponge of miR-140	facilitate the polarization of macrophage towards the M1 subset, promotes angiogenesis and immunosuppressive in HCC	[93]
10.	Non-small cell lung cancer (NSCLC)	GNAS-AS1	24,872	*In vitro* study and clinical study	Patient samples and cell lines; PC9, SPCA1, H358, A549, H1299	miR-4319	GNAS-AS1 down-regulates miR-4319 which directly targets NECAB3. NECAB3 has a role in improving THP-1-differentiated macrophages	promote macrophage M2 polarization and NSCLC cell progression	[94]
11.	Melanoma	Olfr29-ps1	------	*In vitro*, in vivo and clinical studies	Patient samples, different models of mice and cell lines; B16, U937, and HEK 293T	miR-214-3p	Direct sponge miR-214-3p which targets MyD88 to regulate the differentiation and development of MDSCs	Enhance immunosuppressive functions of MDSCs	[95]

**Table 4:** The regulatory role of lncRNAs on miRNAs involved in cancer suppression by different immune pathways

No	Cancer type/origin	Name of lncRNA	HGNC ID number	Model of study	Sample origin	Affected miRNA	Pathway	Immune effect	Ref
1.	Diffuse large B-cell lymphoma (DLBCL)	SMAD5-AS1	6767	*In vitro* study and clinical study	Patient samples and cell lines; TMD8, U2932, GM1287, HEK-293, OCI-Ly3 WSU-FSCCL, JeKo-1, and L428	miR-135b-5p	Downregulation of Wnt/β-catenin pathway via direct competing endogenous and inhibition of miR-135b-5p and activate APC which acts as a negative regulator of Wnt/β-catenin	Suppress lymphoma progression and immune evasion	[60]
2.	Glioblastoma	CASC2c	22,933	*In vitro* study and clinical study	Patients’ samples and cell lines; U251 and GL261 and G1124 and G1104	miR-388-3p	CASC2c and miR-388-3p bound to Factor X and commonly inhibited its expression and secretion.	Decrease M2 subtype macrophage polarization and inhibit glioblastoma multiforme (GBM) growth	[96]
3.	Breast cancer	SNHG1	32,688	*In vitro* study	CD4^+^T isolated from breast cancer patients	miRNA- 448,	Interference of lncRNA SNHG1 up-regulates miRNA- 448, and that decreases the expression of IDO levels in BC	Inhibit the differentiation of Treg cells and reduce immune escape	[55]
4.		XIST	12,810	*In vitro* study and clinical study	Patient samples and cell lines; MCF7, ZR75-1, SKBR3 and MDA-MB231 (MDA231)	exosomal mirRNA-503	Decrease M1-M2 polarization of microglia by upregulating the secretion of exosomal mirRNA-503	Decrease immune suppressive in microglia which decease T-cell proliferation. Also, suppress brain metastasis	[97]
5.	Gastric cancer (GC)	GAS5	16,335	*In vitro* study and clinical study	NK cells isolated from PBMC of GC patients and cell lines; MGC-803 and NK-92	miRNA-18a.	Up-regulation of lncRNA GAS5 in NK cells competes and inhibits miRNA-18a. in addition, increase the secretion of IFN-γ and TNF-α	Increase cytotoxicity and killing effect of NK cells against GC	[98]
6.	Hepatocellular carcinoma (HCC)	FENDRR	43,894	*In vitro* study and clinical study	Patient samples and cell lines; MHCC97, HCCLM3, HepG2, Hep3B, and Huh7	miR-423-5p	LncRNA FENDRR up-regulates GADD45B via direct sponge and inhibits miR-423-5p	Suppress the Treg-mediated immune escape of HCC	[57]
7.	Endometrial cancer	NIFK-AS1	27,385	*In vitro* study and clinical study	Patient samples and cell lines; Ishikawa cells THP-1 cells	miR-146a	lncRNA NIFK-AS1 can target and suppress miR-146a and that promotes the expression of target gene Notch1	Inhibit the M2-like polarization of macrophages, endometrial cancer invasion and progression	[99]

In HCC, overexpression of lncRNA fetal-lethal non-coding developmental regulatory RNA (FENDRR) reduces Treg differentiation, and cell proliferation and induces apoptosis *in vitro* and in vivo by sponging miRNA-423-5p [57]. In contrast, LINC00662 enhanced HCC tumor progression and metastasis in vivo by competitively binding and inhibiting miRNA-15a, miRNA‐16, and miRNA‐107, up regulation of WNTA3 and activation of Wnt/β‐catenin signaling and M2 macrophage polarization [58].

Diffuse large B cell lymphoma (DLBCL) is the most common disorder derived from the B-lymphocytes. LncRNA small nucleolar RNA host gene 14 (SNHG14) induced immune evasion and DLBCL progression by sponging miRNA-5590-3p, increasing the expression of Zinc finger E-box binding homeobox 1 (ZEB1). ZEB1 is a transcriptional factor that functions as an oncogene regulating migration, invasion, EMT, and development of various types of cancer [59].

Meanwhile, lncRNA SMAD5 antisense RNA 1 (SMAD5-AS1) suppressed immune escape and DLBCL progression by directly sponging miRNA-135b-5p, increasing adenomatous polyposis coli gene expression, and suppressing Wnt/β-catenin pathway. This result showed that SMAD5-AS1 could be used as a DLBCL biomarker and treatment target [60].

Interestingly, lncRNA HOTAIR enhances immune escape and tumor progression of both gastric cancer and cervical cancer by promoting the expression of human leukocyte antigen (HLA)-G. HLA-G is a member of the non-classical MHC family and plays a key role in tumor cell escape from host immune surveillance by inhibiting immune cell activities. In gastric cancer, HOTAIR acts as a ceRNA by sponging miRNA-152, resulting in promoting HLA-G expression [61]. Meanwhile, in cervical cancer, HOTAIR triggers the expression of HLA-G by directly sponging and inhibiting miRNA-148a [62].

In summary, both miRNAs and lncRNAs can influence tumor growth, invasion, and metastasis by enhancing the activation of oncogenic pathways and reducing the expression of tumor suppressors, hence regulating tumor growth, invasion, and metastasis. Some examples of lncRNAs-miRNAs interactions which contributed to enhancement of immune escape and tumor progression of different cancer types, include lncRNA SNHG1/ miRNA- 448, lncRNA BCRT1/ miRNA-1303, lncRNA FENDRR/ miRNA-423-5p, lncRNA SNHG14/ miRNA-5590-3p, lncRNA SMAD5-AS1/ miRNA-135b-5p and lncRNA HOTAIR. Also, lncRNA HOTAIR promotes the expression of HLA-G in both gastric and cervical cancer by sponging two different miRNAs; miRNA-152 and miRNA-152, respectively.

## Studying the clinical trials of lncRNAs to find a potential prognostic factor for efficient immunotherapy

The relevance of lncRNA as a predictive factor for cancer cell immunotherapy responsiveness was previously demonstrated in a clinical trial. The data for this study came from TCGA of immunotherapy patients, which included 419 cancer patients. Patients were divided into two groups: (a) 348 patients with bladder cancer from the IMvigor 210 trial phase 2 who were treated with the PD-L1 inhibitor “Atezolizumab,“ and (b) 71 patients with melanoma who were treated with anti-PD-1, anti-cytotoxic T-lymphocyte–associated protein 4 (CTLA4), and cytokine tumor vaccine. Other cancer patients who participated in this study were 493 patients with lung squamous cell carcinoma, 1082 patients with breast cancer, 406 patients with bladder cancer, and 457 patients with melanoma. LASSO was included in the study since since the patients were naive to immunotherapy medications. To expect an appropriate immunotherapeutic response, researchers employed a prediction analysis method that produces models with excellent prediction accuracy. Four of the most significant lncRNAs, AC002116-2, AP000251-1, TMEM147-AS1, and NKILA, have functional immune predictions, according to the findings. Patients with lower levels of these lncRNAs had a higher OS, overall response, and full response length [63].

Another clinical investigation found that lncRNAs have a function in the organization of immunity and the TME in non-small cell lung cancer (NSCLC), which might be useful for prognosis. To differentiate a lncRNA Signature (TILSig) as an indication of immune cell infiltration in patients with NSCLC, researchers developed a unique computational technique . This method was developed using information gathered from an integrated investigation of immune and clinical lncRNA profiles in 115 immune cell lines, 187 NSCLC cell lines, and 1533 NSCLC patients. TILSig divided the patients into two categories: (A) Patients in the high-risk group are immune-cold and have less immune cell infiltration; (B) patients in the low-risk group are immunologically hot and have more immune cell infiltration. Seven TILncRNAs (HCG26, PSMB8-AS1, TNRC6C-AS1, CARD8-AS1, HCP5, LOC286437, and LINC02256) were shown to be associated with immune infiltration in NSCLS patients. Furthermore, patients in the immunological-hot group had a considerably higher survival rate and immune cell infiltration than those in the immune-cold group [64].

Another clinical trial intended to create a lncRNA-based risk signature and nomogram that might predict OS in gastric cancer (GC) patients. The primary cohort consisted of 341 patients with clinical and lncRNA expression data in The Cancer Genome Atlas stomach adenocarcinoma (TCGA STAD), the internal validation cohort consisted of 172 randomly assigned patients, and the external validation cohort consisted of 300 patients from the GSE62254 dataset. Gene set enrichment analysis (GSEA) was also used to investigate the pathway enrichment for the risk signature [65]. The expression patterns of various lncRNAs were also investigated in clinical samples from ten GC patients. The findings indicated a 14-lncRNA signature that was strongly associated with GC patients’ OS and performed well on C-index, the area under the curve, and calibration curve assessments. In univariate and multivariate Cox regression analysis, the lncRNA signature was demonstrated to be an independent predictor of GC patients. Therefore, a nomogram combining the lncRNA signature and clinical factors was developed to predict OS in patients with GC in the original cohort, and it demonstrated high predictive values for survival in the TCGA cohort and the other two validation cohorts. GSEA also observed that the newly identified lncRNAs may affect carcinogenesis and prognosis in GC patients via influencing the autophagy pathway. According to experimental validation, the expression of lncRNAs in clinical samples and the STAD dataset followed the same pattern. Both the risk signature and the nomogram are excellent prognostic markers for GC patients, according to these studies [65].

## Current challenges and future perspectives

This review mainly focuses on the crosslinking between lncRNAs and TME which affects the response of cancer patients to immunotherapy. Immunotherapy has become one of the most well-established treatment options for a wide range of cancers. The most recent forms of immunotherapies with excellent outcomes, notably in hematologic malignancies, are CPIs and CAR-T-cells [66]. Many changes and mutations in cancer cells as well as other stromal cells in the tumor microenvironment can lead to immune escape and resistance. Immune-related lncRNAs play a key role in the regulation of immunological cell-specific gene expression, which modifies immune processes by controlling the environment and activities of immune cells as well as anti-inflammatory substances [1]. LncRNAs have the features of tissue-specific expression. They are characterized by relative stability in circulating body fluids, which make lncRNAs useful as cancer biomarkers and facilitate non-invasive detection. Immunotherapy based on lncRNAs has numerous advantages. LncRNAs can regulate a number of downstream target genes by participating in a variety of cell signalling pathways, which can help to control cancer treatment. Furthermore, many regulatory sites of lncRNAs can interact and interfere with other molecules, facilitating the development of new structure-based anticancer medicines [67].

More importantly, lncRNAs can also influence the interaction between CSCs and immune cells in the tumor microenvironment, potentially resulting in CSC resistance to immunotherapy and increased tumor growth and progression. Immunological-related lncRNAs might influence immune responses either directly by influencing adjacent protein-coding genes or indirectly by sponging miRNAs through several pathways [68]. Finally, we sought to outline and emphasize the role and levels of expression that have been linked to immune cell formation, differentiation, and activation, which may influence immunotherapeutic responses for a variety of malignancies and other disorders. Future research may be required to elucidate the many processes and pathways of immune-related lncRNAs in the tumor microenvironment, which might influence cancer development and progression.

## Conclusion

LncRNAs play a key function in modifying the TME and controlling tumor cell immune escape. As a result, lncRNA-based targeted cancer immunotherapy has a bright future ahead of it. Despite the ongoing issues with lncRNA-based therapy, as research advances and becomes more refined, the use of lncRNA as a therapeutic target will contribute to the development of novel cancer therapeutic techniques.

## Abbreviations

LncRNA: long noncoding RNAs.
CI: Cancer immunotherapy.
APC: antigen –presenting cells.
mAbs: monoclonal antibodies.
CAR-T cell: antigen receptor-T cell.
ICIs: immune checkpoint inhibitors.
m7G: 7-methyl guanosine.
ORFs: open reading frames.
TLR: Toll-like receptor.
ceRNAs: competing endogenous RNAs.
miRNAs: microRNAs.
RNP: ribonucleoprotein.
ALL: Acute lymphoblastic leukemia.
INSR: Insulin receptor.
Treg: T regulatory cells.
CTLs: cytotoxic T lymphocytes.
IME: immune microenvironment.
Foxp3: forkhead box P3.
NK: natural killer.
TCL6: T-cell leukemia/lymphoma 6.
PR: Progesterone receptor.
DCs: Dendritic cells.
TILs: tumor-infiltrating lymphocytes.
TAMs: tumor-associated macrophages.
ROS: reactive oxygen species.
MDM2: Mouse double minute 2 homolog.
Cox-2: Cyclooxygenase-2.
HCC: hepatocellular carcinoma.
EGFR: epidermal growth factor receptor.
c-CBL: c-Casitas B-lineage Lymphoma.
ERK1/2: extracellular signal-regulated kinases 1/2.
AP-1: Activator protein 1.
TCGA: The Cancer Genome Atlas.
CGGA: Chinese Glioma Genome Atlas.
OSs: overall survivals.
RS: risk score.
IRLS: immune-related lncRNA signature.
TME: tumor microenviroment.
Pvt1: plasmacytoma variant translocation 1.
MDSCs: Myeloid-derived suppressor cells.
CSCs: cancer stem cells.
PGE2: prostaglandin E2.
EMT: epithelial–mesenchymal transition.
LGL: large granular lymphocytes.
HAL: Hypoxia-associated lncRNA.
RUNX1-IT1: runt-related transcription factor 1 Intronic Transcript 1.
lnc-DILC: LncRNA down-regulated in liver cancer stem cells.
GSCs: glioma stem cells.
SNHG1: small nucleolar RNA host gene 1.
IDO: Indoleamine 2,3-dioxygenase.
BCRT1: breast cancer-related transcript 1.
PTBP3: Poly pyrimidine tract-binding protein 3.
FENDRR: fetal-lethal non-coding developmental regulatory RNA.
DLBCL: Diffuse large B cell lymphoma.
ZEB1: Zinc finger E-box binding homeobox 1.
SMAD5-AS1: lncRNA SMAD5 antisense RNA 1.
HLA: human leukocyte antigen.
NSCLC: non- lung small cell cancer.
GSEA: Gene set enrichment analysis.
